# Editorial

**Published:** 1898-05

**Authors:** 


### Editorial.

### THE TRUTH ABOUT CIGARETTES.

It has been the fashion for years for the laity and profession alike to deprecate the habit of cigarette smoking mildly or to consign its habitues to the limbo of the unclean. That such charges against the cigarette were founded upon very insecure premises was known to every one who had observed the effect of the habit of smoking them upon his own person or upon others. He knew that in this form he got the minimum amount of tobacco, and, therefore, argued unhomeopathically, that he received the minimum effect. No one prior to W. H. Garrison, Esq., has hitherto had the temerity to combat the popular notion, but in his "Brief for the Cigarette" he has done it so well that many are already persuaded that they have been in error as to the harmfulness of the practice. The Hon. Clark Bell has published a pamphlet containing this paper and also the expressions of opinion from eminent physicians throughout the country. There is a singular unanimity of opinion from them that there is but little truth in the general clamor against the cigarette, except in the case of the young, when, of course, the habit is necessarily detrimental. It is very refreshing to see a long-standing, almost, through age, respectable, lie so cleverly nailed, and it is to be hoped that the work of these gentlemen will give a permanent set-back to another sanctified fraud.

---

The Record desires to tender its sympathies to Dr. V. O. Hardon in his deep affliction in the loss of his young and beautiful wife.

---

The Charles Roome Parmele Company have issued an attractive pamphlet, "Arsenauro and Mercauro," containing clinical records as reported in medical journals and covering the wide range of utility of these elegant and effective preparations.

---

The Dios Chemical Company, of St. Louis, are remembering their friends with a neat glass paperweight, the lower surface of which is a mirror by means of which the doctor may preen his plumes between patients.

